# Determination of Cyclopropane Fatty Acids in Food of Animal Origin by ^1^H NMR

**DOI:** 10.1155/2018/8034042

**Published:** 2018-04-01

**Authors:** Veronica Lolli, Angela Marseglia, Gerardo Palla, Emanuela Zanardi, Augusta Caligiani

**Affiliations:** Department of Food and Drug, University of Parma, Parco Area delle Scienze 27A, 43124 Parma, Italy

## Abstract

Cyclopropane fatty acids (CPFAs) are unusual fatty acids of microbial origin, recently detected in milk and dairy products. CPFAs have been demonstrated to be interesting molecular markers for authentication of dairy products obtained without ensiled feeds. Moreover, they can also be recognized as a new secondary component of human diet. Information is lacking on the presence of cyclic fatty acids in other food sources. Cyclopropane fatty acids have been detected by GC-MS analysis in cheese and other animal fats in concentration ranging from 200 to 1000 mg/kg fat, but in some cases, the complex fatty acid profile and the possible presence of interfering peaks make the separation not straightforward and the quantification uneasy. Therefore, a new reliable ^1^H NMR method was developed to detect and measure CPFA content in different foods of animal origin, based on the detection of the characteristic signals of cyclopropane ring. The ^1^H NMR (600 MHz) method showed detection limits comparable with those of full scan GC-MS, and it allowed the identification and quantitation of the cyclopropane fatty acids in different foods.

## 1. Introduction

Cyclopropane fatty acids are unusual fatty acids found in microorganisms, both Gram-negative and Gram-positive, and seed oils of some tropical plants and protozoa [1, 2]. The bacterial production of cyclopropane ring is related to changes in the membrane fatty acids composition and represents one of the most important adaptive microbial responses that favours the stress tolerance of several bacteria, such as *Lactobacillus helveticus*, *L. bulgaricus*, *L. acidophilus*, and *L. sanfranciscensis* [1].

In plants, CPFAs are usually minor components, where cyclopropene fatty acids are the most abundant. They are present in Malvaceae, Sterculiaceae, and Sapindaceae, representing a significant component of *Litchi chinensis* and *Sterculia foetida* seed oils, principally sterculic acid [2].

**Figure 1:**
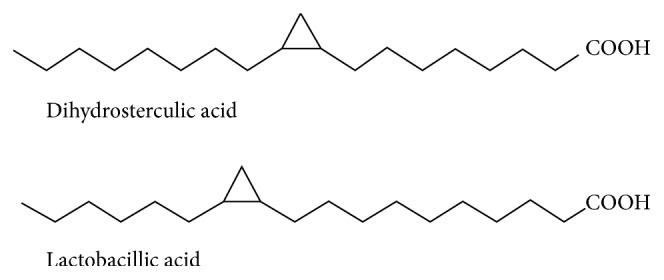
Main cyclopropane fatty acids detected in dairy products.

Recently, we identified by GC-MS the presence of CPFAs (dihydrosterculic and lactobacillic acids, Figure 1) in milk and dairy products [3, 4] and more recently in meat (unpublished results).

Due to the undoubtable importance of these foodstuffs in human diet, it appears clear that a deep investigation on the dietary intake of these fatty acids and their effects on humans gains importance.

CPFAs have been also recently identified in human serum and adipose tissue [5], suggesting that they are absorbed as the other fatty acids and can exert physiological effects. Moreover, CPFAs are minor fatty acids but their presence in milk fat is in the hundred-ppm order [4], so their dietary intake may be not negligible.

CPFAs (mainly dihydrosterculic acid) also play an important role in food authentication: in fact, they were discovered in milk and dairy products from cows fed with silages, and their determination has been demonstrated to be a powerful tool for the authentication of Protected Denomination of Origin (PDO) cheeses, such as Parmigiano Reggiano, where the use of silages in cow feeding is forbidden [6]. In this context, “Consorzio del Formaggio Parmigiano Reggiano” has proposed a modification on the Production Specification Rules, including the determination of CPFAs among the official controls (UNI 11650).

Therefore, CPFAs represent an almost completely new field of research in food lipids and it is important to develop different methods of detection and quantification, in view of an expected growing body of research, both in food characterization/authentication and in food safety aspects.

Gas chromatography methods currently dominate the literature for the determination of main and secondary fatty acids in foods [7–9], and we previously applied this technique for the qualitative and quantitative determination of CPFAs in milk and dairy products [3, 4, 6]. However, gas chromatography analysis requires time-consuming sample derivatization with the risk of interfering by-products and use of large amount of solvents [10]. Moreover, in the particular case of fat from animal origin, the extreme complexity of fatty acid profile makes the separation and quantification of minor fatty acids a challenging issue. For example, more than 400 different fatty acids were detected in milk [11]. In the case of cyclopropane fatty acids, we obtained its separation in cheese fat by using apolar capillary column [6]; however, this column is not suitable for the optimal separation of fatty acids, so it is possible that changing the food matrix interferences occur. It is also possible that other cyclopropane fatty acids were present but undetectable because they were overlapped by the most abundant fatty acid signals. So, it is important to have an alternative method to confirm the cyclopropane ring presence and possibly to correctly quantify CPFAs. Moreover, the development of a rapid method that provides the necessary analytical information with minimal sample preparation would be advantageous. NMR spectroscopy is one such analytical tool that avoids sample derivatization and offers the benefit of short data acquisition times.

Nuclear magnetic resonance spectroscopy has started to represent an interesting tool to analyse biofluids and food and beverages, and in the case of lipids, it represents a reliable and fast alternative to traditional methods such as gas chromatography. This was due to the advantages of this technique as the simplicity of the sample preparation (usually it only requires the fat dissolution in deuterated chloroform) and measurement procedures, the instrumental stability, the increase of sensitivity, and modern pulse sequences, with simultaneous suppression of big signals [12].

For these reasons, the use of NMR spectroscopy has established a significant role in the analysis of lipids [13]. Several studies consider the analysis by ^1^H NMR of triacylglycerol composition as a useful tool for both triglyceride quantitation and sample classification [14]. Minor fatty acids were also object of investigation by NMR, especially conjugated linoleic acids (CLAs) [15].

NMR could represent an ideal method to detect CPFAs due to the characteristic signals of the protons of the cyclopropane unit between −0.30 and −0.35 ppm [16], which permit their detection in a zone of ^1^H NMR spectrum practically free from other signals. This highly shielded position of cyclopropane resonance is conventionally explained by the anisotropy of the C–C bond, just opposite to CH_2_ group in a three-membered ring, or by an aromatic-like ring current involving the six electrons in the three C–C bonds (*σ* aromaticity) that shields cyclopropane protons [17].

Therefore, with the aim to investigate on the presence of CPFAs in foods, we developed a new fast and reliable quantitative ^1^H NMR method, to be used as alternative to gas chromatographic methods and to confirm the presence of CPFAs in foods.

## 2. Experimental

### 2.1. Materials

Methanol, *n*-hexane, dichloromethane, trimethylchlorosilane, hexamethyldisilazane, 1-decanol, sodium sulphate anhydrous, sodium carbonate, deuterated chloroform, and tetracosane were from Sigma-Aldrich (Saint Louis, MO, USA), and hydrochloric acid and potassium hydroxide pellets were from Carlo Erba (Milan, Italy). Dihydrosterculic acid methyl ester was from Abcam (Cambridge).

All the solvents, standards, and reagents were of analytical grade.

Cheese, meat samples from several species animals, cured meat, and commercial fish were analysed for the content of cyclopropane fatty acids. Most of them were purchased from the market (Parma, Italy). Samples of cheese and meat produced without ensiled feeds were kindly provided from Parmigiano Reggiano Cheese Consortium and Prof. Riccardo Bozzi of the University of Florence, respectively.

### 2.2. Fat Extraction

Lipid extraction following the Folch method [18] was performed. 10 g of sample was homogenized with 75 mL of dichloromethane : methanol (2 : 1, v/v). The mixture was centrifuged (10 min, 3000 rpm) and filtered. This procedure was repeated three times. The three filtrates were transferred to a graduate cylinder, and a volume of about 50 mL KCl 0.88% in distilled water was added. The mixture was shaken vigorously. The final biphasic system was decanted, and the upper aqueous phase was eliminated. The lower organic phase was filtered through anhydrous sodium sulphate and collected. Lipid content was then recovered after solvent was evaporated with a rotary evaporator under vacuum.

### 2.3. ^1^H NMR Analysis

#### 2.3.1. Synthesis of Internal Standard Trimethylsilyl Decanol (TMSD)

0.2 mL of 1-decanol, 0.3 mL of trimethylchlorosilane, and 0.6 mL of hexamethyldisyiazane were mixed in a screw cap septum vial. Mixture reacted for 1 h at 60°C, neutralized with sodium carbonate, and then dried with anhydrous sodium sulphate. Reaction mixture was diluted with 1 mL of hexane, filtered, taken to dryness in a rotary evaporator, and the residue weighed. Purity of trimethylsilyl decanol (TMSD) was confirmed by ^1^H NMR and by GC-MS analysis in the conditions reported in Section 2.4.

#### 2.3.2. Preparation of CPFA and TMSD Standard Solutions

Appropriate amounts of trimethylsilyl decanol (TMSD, internal standard) and CPFAs were weighed and added separately to CDCl_3_ (10 mL) to yield two final stock solutions of about 500 mg/L each.

Adequate amounts of CPFA and TMSD stock solutions were transferred in 5 mm NMR tubes and taken to the final volume of 1 mL with CDCl_3_ to obtain working solutions at 100, 50, 25, and 5 *µ*g/mL of CPFAs, all containing 10 *µ*g/mL of TMSD.

#### 2.3.3. Preparation of Spiked Samples

100 mg of meat fat (chicken) and cheese fat (Parmigiano Reggiano) both negative to CPFAs were spiked with the appropriate amount of CPFA and TMSD solutions and taken to the volume of 1 mL of CDCl_3_ to obtain the same final concentrations reported above for standard solutions.

#### 2.3.4. ^1^H NMR Acquisition

100 mg of fat was dissolved in 1 mL of CDCl_3_ containing 0.01 mg of TMSD as internal standard. ^1^H NMR spectra were recorded on a Varian INOVA-600 MHz spectrometer (Varian, Palo Alto, CA, USA), equipped with a 5 mm triple resonance inverse probe. Data were collected at 298 K, with 32 K complex points, using a 90° pulse length. 1024 scans were acquired with an acquisition time of 1.707 s and a recycle delay of 2 s. Presaturation of the fatty acids –CH_2_– signal (1.25 ppm) was performed in order to assure a correct digitization of small signals as CPFAs. The NMR spectra were processed by MestReC software 6.0.2 (Santiago de Compostela, Spain, EU): spectra were Fourier transformed with FT size of 64k and 1 Hz line-broadening factor, manually phased and carefully baseline corrected, and referenced to the chloroform signal (7.26 ppm). Baseline correction was further manually optimized in the zone of interest (from −1 ppm to 0.7 ppm).

#### 2.3.5. Quantitative Analysis

CPFA concentrations were obtained by integrating the peak area of the ^1^H NMR signal at −0.35 ppm and the methyl signal of the trimethylsilyl group of the internal standard (TMSD) at 0.1 ppm.

The CPFA integral was converted in mass value (mg) according to the following formula, as previously reported [19]:

(1) $$\begin{array}{c} \mathrm{ACPFA}\times \frac{\mathrm{EWCPFA}}{\mathrm{mg}\;\mathrm{CPFA}}=\mathrm{ATMSD}\times \frac{\mathrm{EWTMSD}}{\mathrm{mg}\;\mathrm{TMSD}}, \end{array}$$

where ACPFA = spectral area of CPFA, ATMSD = spectral area of internal standard, EWCPFA = equivalent weight of the analyte, EWTMSD = equivalent weight of internal standard, and EW = (molecular weight/number of hydrogens in the signal).

Absolute amount of CPFAs obtained was finally expressed as mg/kg of fat.

#### 2.3.6. Linearity and Limit of Detection and Quantification

The limit of detection (LOD) and the limit of quantification (LOQ) were calculated utilizing the *S*/*N* ratio methods, based on the determination of the peak-to-peak noise [20]. LOD and LOQ were, therefore, calculated as the concentrations of CPFAs producing a recognizable peak with a signal-to-noise ratio of, respectively, 3.3 and 10. LOD and LOQ were determined both in pure standard solution and in a sample of meat fat negative to CPFAs spiked with different concentrations of CPFAs.

#### 2.3.7. Accuracy, Precision, and Recovery of the Method

The accuracy of the CPFA recovery was determined by assaying samples with known concentrations of CPFAs, both as pure compounds and as spiked matrix. The precision was expressed as coefficient of variation (CV%). Recovery of analytes was determined by spiking sample of fat free from CPFAs with pure dihydrosterculic acid.

### 2.4. Gas Chromatographic Analysis

GC-MS quantitative analysis was performed as previously reported [6]. Briefly, 200 mg of fat was dissolved in hexane (5 mL) and mixed for 1 min with 0.2 mL of KOH 10% (Carlo Erba, Milan, Italy) in methanol. After phase separation, the superior organic phase was added to internal standard (tetracosane) and injected (1 *µ*L, split mode) on an Agilent Technologies 6890N gas chromatograph (Agilent Technologies, Palo Alto, CA, USA) coupled to an Agilent Technologies 5973 mass spectrometer (Agilent Technologies, Palo Alto, CA, USA). A low-polarity capillary column (SLB-5ms, Supelco, Bellafonte, USA) was used. The chromatogram was recorded in the scan mode (40–500 *m*/*z*) with a programmed temperature from 60°C to 280°C.

## 3. Results and Discussion

### 3.1. CPFA Signal Detection

**Figure 2:**
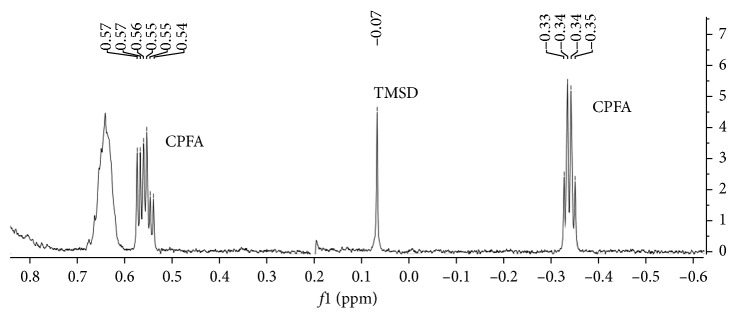
^1^H NMR spectrum (600 MHz, CDCl_3_) of dihydrosterculic acid standard and trimethylsilyl decanol (TMSD) in the very upfield region of the spectrum. The CPFA signal at −0.34 ppm and the TMSD signal at 0.07 ppm were selected for quantification.

Figure 2 depicts the characteristic upfield zone of ^1^H NMR spectrum of dihydrosterculic acid and TMCD. ^1^H NMR shows two individual peaks at −0.35 and 0.60 ppm for the methylene protons of the cyclopropane ring. Assignments were previously made by Knothe [16] with the aid of 2D correlations: the upfield signal is assigned to the *cis*-proton and the downfield signal to the *trans*-proton. The two methine protons of the cyclopropane ring are located at 0.68 ppm. The other CPFA proton signals are located at lower fields; for example, the four protons in alpha position with respect to the cyclopropane ring display a distinct shift at 1.17 ppm and the signal of the other two protons is observed at 1.40 ppm, within the broad methylene peak [16]. Among all these specific signals, the *cis*-methylene proton of the cyclopropane ring can be easily assigned and used for quantification because it does not overlap with any other signal of fatty acids that could be observed in a complex food lipid ^1^H NMR spectrum.

Because the integral of a given peak in a ^1^H NMR spectrum is directly proportional to a corresponding number of resonant nuclei, peak areas of CPFA *cis*-methylene proton can be compared with the peak area of TMSD trimethylsilyl group close to 0.1 ppm and used for the determination of total CPFA content. This internal standard was specifically synthetized starting from a medium chain linear alcohol as decanol because it is not volatile, soluble in apolar solvents, and its trimethylsilyl group gives a singlet close to the CPFA selected signal. Tetramethylsilane (TMS) was commercially available but it cannot be used as quantitative internal standard due to its volatility. The synthesis of TMSD was quick and simple with a high yield (about 80%). The purity was determined by GC-MS and ^1^H NMR (data not shown).

^1^H NMR spectra of food fats are characterized by a dominating fatty acid methylene group peak at 1.2 ppm that is many orders of magnitude greater than those of the component of interest. This causes a number of problems: first of all, it prevents a correct digitization of small signals, hampering their observation and quantification, but the tail of this large signal can also determine a distortion of the baseline in the zone of CPFA chemical shifts. Therefore, a suppression of this signal was performed during spectra acquisition.

### 3.2. Quality Parameters of ^1^H NMR Analysis

The quantitative ^1^H NMR method was developed with the aim to determine CPFA concentration in a broad range of food fats, in particular in fats of animal origin (dairy products, meat, and fish). As a first step, the method was subjected to validation in terms of precision, accuracy, linearity, detection, and quantitation limits, following recommendations of the International Conference on Harmonization (ICH 2005: Validation of analytical procedures: text and methodology. Harmonized tripartite guideline, Q2, R1). The validation tests were performed on pure solutions of CPFA (dihydrosterculic acid) and on cheese and meat matrices naturally free from CPFAs, spiked with dihydrosterculic acid as reported in Experimental.

**Figure 3:**
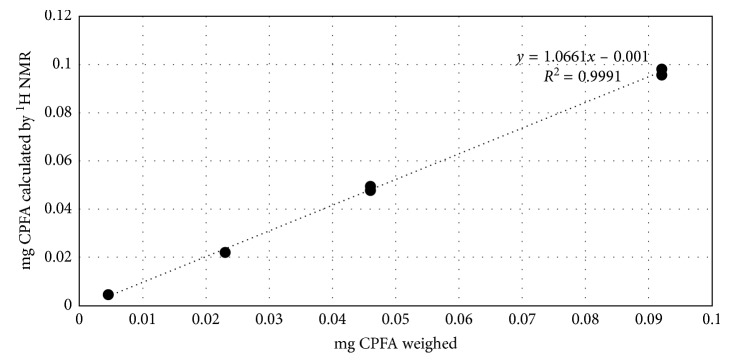
Regression curve for CPFA standard solution measured with the ^1^H NMR method.

To determinate accuracy and precision, solutions containing weighed amount of CPFAs were analysed by ^1^H NMR in the experimental conditions previously reported. Measured results for the standard solutions were in agreement with the amounts weighed in the range of concentrations of 0.005–0.1 mg/mL of CPFA, a range that corresponds with the final intube concentration of the analytes in real samples of fat. The linearity was demonstrated in the same range (Figure 3). The limit of detection and the limit of quantification were calculated utilizing the *S*/*N* ratio method described above. LOD and LOQ were calculated in pure standard solutions. The instrumental quantification limit for CPFA standard (LOQ, signal to noise ratio higher than 10) in the experimental conditions reported was about 0.01 mg/mL while the limit of detection (LOD) was obtained at 0.0025 mg/mL (*S*/*N* ratio 4). The ^1^H NMR (600 MHz) method developed showed detection limits in pure standard solutions comparable with those generally achieved by full scan GC-MS. Coefficients of variation (CV%) for three replicate measurements of each standard concentration were lower than 3%, indicating a good precision of the method.

**Figure 4:**
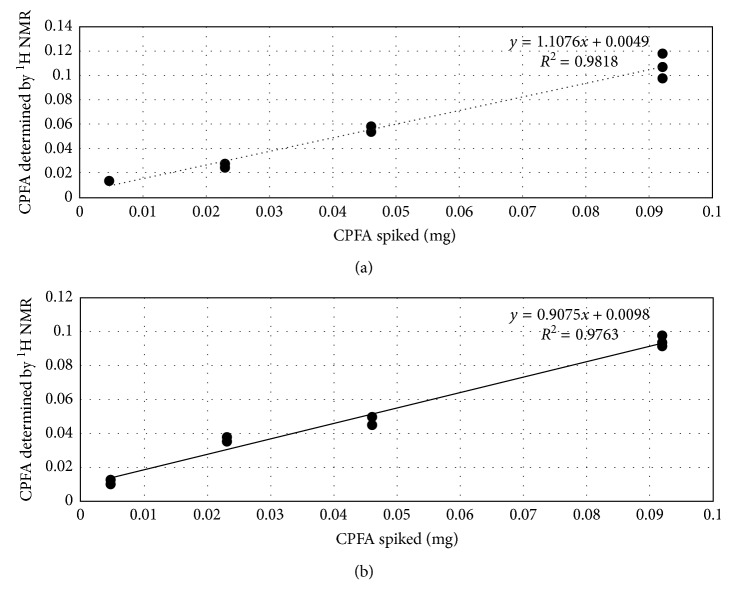
Calibration curves of CPFA spiked matrices: (a) cheese and (b) chicken meat.

Linearity, LOD, and LOQ were also calculated in two different matrices, cheese and chicken fat. The samples chosen for spiking were previously analysed by GC-MS and ^1^H NMR and were found negative to CPFAs. Each matrix was spiked with four different amounts of dihydrosterculic acid as reported in Experimental. Regression curves obtained are shown in Figure 4.

The linearity is maintained, as in the case of pure standard; however, in both cases, the intercept of the regression curve indicates a matrix effect, most pronounced for chicken fat. The limit of quantification for cheese was 120 mg/kg fat (*S*/*N* ratio of 10), and the limit of quantification was found to be 50 mg/kg fat (*S*/*N* ratio of 10). In the case of chicken meat, LOQ and LOD were 180 mg/kg fat and 70 mg/kg fat, respectively. Comparing these values with those obtained for pure standard solutions, results demonstrate not negligible matrix effect both in cheese and in meat fat, suggesting that an external calibration in a fat matrix is needed for an accurate quantification. On the contrary, the method can be easily applied for a rapid semiquantitative and qualitative analyses in both matrices.

### 3.3. Application to Real Samples

#### 3.3.1. Comparison of ^1^H NMR-Based Method and GC-MS Method

**Figure 5:**
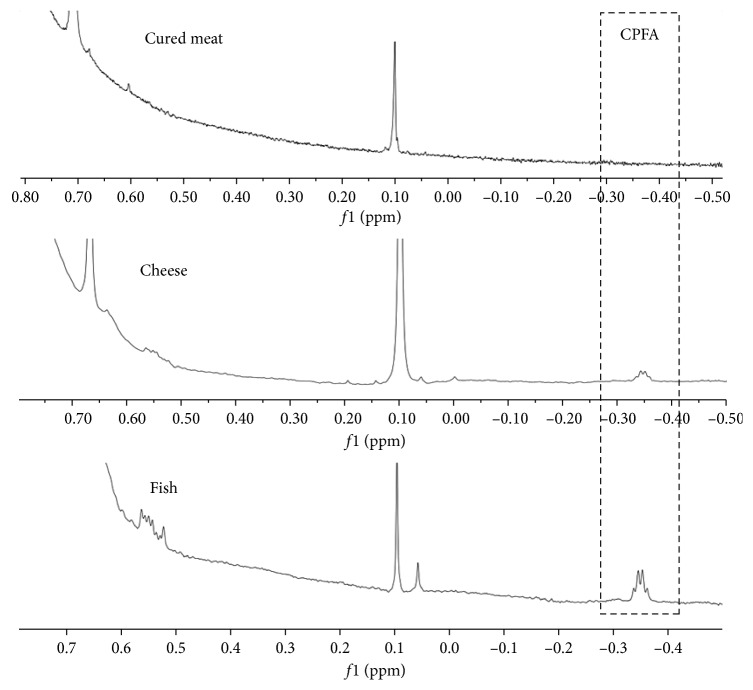
Enlargement of ^1^H NMR 600 MHz spectra in the zone from −0.5 to 0.8 ppm, showing the signal of the cyclopropane ring (at −0.35 ppm) used for CPFA quantification in cured ham (negative to CPFAs) and cheese and fish fat (positive to CPFAs).

Different samples of meat, fish, and cheese were analysed both by ^1^H NMR and GC-MS. GC-MS quantitative analysis was performed based on the method previously applied for cheese, as described in Caligiani et al. [6].  Figure 5 shows the enlargement of the diagnostic region for CPFAs in the ^1^H NMR spectra of lipids extracted from cheese, meat, and fish (containing the TMSD internal standard), confirming that the signal does not overlap with any other resonances representative of fatty acids.

Table 1 reports the list of the samples analysed for each food category, the number of samples negative or positive to CPFAs, and the comparison between GC-MS and ^1^H NMR results. A reference Grana Padano cheese was specifically analysed both by GC-MS and ^1^H NMR method to demonstrate the accuracy of the new ^1^H NMR method. Then, five samples of Parmigiano Reggiano and five samples of Grana Padano were tested because in the case of cheese, we had collected previously many data confirming the association between the use of ensiled feeds and the presence of CPFA [4, 6]. ^1^H NMR analysis confirmed the positivity at CPFA for all samples of Grana Padano (ensiled feeds allowed) and the negativity of all Parmigiano Reggiano samples (ensiled feeds forbidden), indicating that ^1^H NMR could be an attractive alternative technique to GC-MS to assure the authenticity of Parmigiano Reggiano and other cheeses forbidding the use of ensiled feeds in their disciplinary of productions. In the case of cheese, quantitative results suggested very good agreement between data obtained by the quantitative ^1^H NMR analysis and previous GC analysis method.

Concerning meat, CPFAs were detected in the GC-MS profiles of most of the commercial bovine meat samples in concentrations varying from 100 to 400 mg/kg of the total fat. CPFAs were detected by ^1^H NMR analysis in all commercial bovine meat samples previously resulted positive by the GC-MS analysis, with good agreement of the quantitative results. CPFAs were absent in two samples of certified meat from cows not fed with fermented forages, and this was evidenced by both techniques. The GC-MS analysis of other meat samples (pork and chicken) was negative to CPFAs both in GC-MS and ^1^H NMR method. In the case of pork cured meat (salami and ham), the GC-MS analysis showed the presence of a signal at the retention time of CPFAs with concentrations of 60–100 mg/kg of the total fat. However, the corresponding analysis by ^1^H NMR did not show the presence of cyclopropane ring, indicating the presence of an interfering peak in the GC-MS conditions adopted. This interfering peak was also resistant to oxidation as a saturated fatty acid, but it has not been identified yet and it was not easy to obtain a better separation varying chromatographic conditions. Therefore, in the case of pork, cured meat seems to be important to have the NMR confirmation of CPFA presence.

And in cured meat, the GC-MS analysis of fish samples generally showed the presence of interfering signals at the same retention time of cyclopropane fatty acids with the same corresponding mass spectrum (278 *m*/*z*), probably due to the presence of different isomers of nonadecenoic acid. Moreover, lactobacillic acid coeluted with another interfering substance with the corresponding mass spectrum of 165 *m*/*z*. This interfering peak was not resistant to oxidation and it has been suggested to be a furan fatty acid as discussed elsewhere [21, 22].

Therefore, GC-MS analysis alone was not able to confirm the presence or absence of CPFAs in fish samples, but it always required ^1^H NMR analysis. Moreover, observing the preliminary results showed in Table 1 on three fish samples, it seems that GC-MS underestimates the content of cyclopropane fatty acids, suggesting that besides cyclopropane fatty acids with 19-carbon atom skeleton, such as dihydrosterculic and lactobacillic acids, it is possible that other CPFAs with different chain lengths occur in fish.

## 4. Conclusion

A new quantitative ^1^H NMR method was developed for the determination of CPFA content in different food matrices, including dairy products, meat, and fish.

The new method reported here provides absolute quantities of CPFA (mg/kg of total fat) and shows a limit of detection comparable with those of full scan GC-MS. A complete and reliable sample analysis can be performed quickly and requires little sample preparation, reagents, and solvents. This was possible because the CPFA signal was very well defined and did not overlap with others. The role of NMR seems to be most important in meat and fish characterization because the GC-MS analysis was not able to confirm the presence of CPFAs in all the analysed samples due to the presence of interfering peaks.

Results suggested that the NMR analysis approach has potential application as a screening for quantifying cyclopropane fatty acids in meat and fish, as markers of quality and the preliminary data on few meat and fish samples presented here suggest some possible developments. For example, in the context of food authentication, cyclopropane fatty acids might be proposed, as in the case of cheese, as markers of silage feedings are able to authenticate high-quality costly meat whose producers declare the absence of silages in the feeding. This will require the construction of a robust database of meat certificated for the feeding system. This approach could also be extended to fish, to eventually distinguish farmed from wild fish.

Moreover, in the case of fish, NMR method is able to detect a higher amount of CPFAs with respect to GC-MS, indicating an important role of NMR when dietary intake of cyclopropane fatty acid has to be assessed.

## Figures and Tables

**Table 1:** Comparison of ^1^H NMR and GC-MS results on the presence of CPFAs in some representative samples of fat of animal origin.

Samples	Number of analysed samples	CPFA (GC-MS) (mg/kg fat)	CPFA (^1^H NMR) (mg/kg fat)
*Cheese*			
Reference cheese		600 ± 50	690 ± 60
Parmigiano Reggiano	5	<LOD	<LOD
Grana Padano	5	400–700	400–800
*Meat*			
Commercial bovine meat	5	200–400	300–400
Bovine meat of certified origin (not fed with silages)	2	<LOD	<LOD
Other meats (Pork and chicken)	4	<LOD	<LOD
*Cured meat*			
Salami	1	60	<LOD
Parma ham	1	120	<LOD
Bresaola	1	340	400
*Fish*			
Eel	2	180–350	400–590
Mullet	1	120	800
